# Cutaneous leishmaniasis in Tabuk, Saudi Arabia: epidemiological trends from 2006 to 2021

**DOI:** 10.11604/pamj.2023.45.11.38632

**Published:** 2023-05-04

**Authors:** Raafat Abdel Moneim Hassanein, Adel Galal El-Shemi, Bader Mohammed Albalawi

**Affiliations:** 1 Department of Laboratory Medicine, Faculty of Applied Medical Sciences, Umm Al-Qura University, Makkah, Saudi Arabia,; 2 Department of Zoonoses, Faculty of Veterinary Medicine, Assiut University, Assiut, Egypt,; 3 Department of Laboratory and Blood Bank, Alwajh General Hospital, Tabuk, Saudi Arabia

**Keywords:** Cutaneous leishmaniasis, epidemiological trends, Tabuk, Saudi Arabia

## Abstract

**Introduction:**

cutaneous leishmaniasis (CL) is a vector-borne protozoan skin disease that affects all human ages and can pose extreme social and psychological impacts. This study aimed to reveal the epidemiological trends of CL in the Tabuk region, the Kingdom of Saudi Arabia (KSA), during the period from 2006 to 2021.

**Methods:**

patients with CL, who were detected and registered at the regional Vector-borne Diseases Control Unit of the Tabuk province, between January 2006 and December 2021, were analyzed in this retrospective study. The patients´ data included their nationality, gender, and age, and their annual and month-by-month recorded patterns.

**Results:**

a total of 1575 CL patients were reported during the said period. They were 53.1% Saudis and 46.9% non-Saudi expatriates with a ratio around 1.1: 1.0; and they were re-categorized as 83.17% males and 16.83% females with a ratio of 4.9: 1.0 (p <0.5). Additionally, the majority (1002/1575; 63.6%) of these CL patients were in age group of 15-45 years (p <0.5), and the lowest number was in age group of <5 years. Most importantly, there was a continuous annual and month-by-month record of these patients; reflecting CL endemicity in the Tabuk region of KSA.

**Conclusion:**

the present findings imply that CL is endemic in the Tabuk region of KSA. As there is a recent increase in human immigration to this region, sustainable monitoring of CL and improving its control measures is warranted.

## Introduction

Leishmaniasis is a complex vector-borne disease caused by an obligate intracellular flagellated protozoan parasite of the family Trypanosomatidae, order Kinetoplastida, and genus *Leishmania*. There are more than twenty different *Leishmania* species known to infect humans, and their transmission is carried out by the bite of the infected female phlebotomine sandflies [1,2]. Epidemiologically, leishmaniasis is a widespread disease in the tropical and subtropical areas, and it is endemic in nearly 98 countries with a predominant endemicity in the Americas, the Middle East, Central Asia, and the Mediterranean region [3,4]. Annually, more than 70,000 leishmaniasis-related deaths are estimated to occur worldwide [5,6], and up to 1 billion people are currently living at risk of infection in their endemic areas [7].

Clinically, *Leishmania* species can infect all human ages and present multiple manifestations [8]. Depending on the immune and nutritional statuses of the individuals, the leishmanial diseases range from cutaneous leishmaniasis (CL); which encompasses multiple popular, nodular and ulcerative skin lesions, to destructive mucocutaneous ulceration and potentially fatal visceral syndromes [9-11]. Globally, CL is the most common clinical form of leishmanial diseases, and the incidence rate of its new cases is estimated to be over 1.5 million per year [7,12-14]. Moreover, CL can cause serious skin ulcerative lesions with extreme social and psychological impacts, and given low treatment coverage and the unavailability of preventative vaccines, further increases in its global burden and health adverse impacts is mightily anticipated [15]. As a consequence, the World Health Organization (WHO) has lately listed leishmaniasis, particularly its CL form, as one of the most neglected infectious diseases worldwide for which the sustainability of monitoring disease trends, effective surveillance systems, and enhanced control programs is a priority, particularly in countries with known history of leishmaniasis endemicity [16,17]. At that regard, the first case of CL was reported in KSA in 1976 [18] and since that time, great efforts, including an establishment of leishmaniasis control program (LCP) in 1978, are continuing to be made by the Saudi health authorities. In despite, the incidence rate of leishmaniasis, particularly its CL form, continues to be one of the major health problems in KSA [19-21]. The present study was therefore designed to identify the epidemiological trends of CL over the past period of 2006 to 2021 in the Tabuk region, KSA.

## Methods

**Study design and setting:** the present retrospective study was designed to reveal the epidemiological trends related to CL infection among individuals who were resident in the Tabuk region, KSA, during the period from January 2006 to December 2021. An ethical approval (IRB # TU-077-022-127) was obtained from the Institutional Review Board of Health Affairs in the Tabuk province, and patients with confirmed CL during the said period, and they were registered at the Vector-borne Diseases Control Unit of the Tabuk province, were included here. Following the guidelines of the established vector-borne diseases control program by the Saudi Ministry of Health (MoH), the diagnosis and treatment of any vector-borne positive case, including leishmaniasis, is totally hospital-based, and case information are directly delivered to the Vector-borne Diseases Control Unit of this region through a specific health program axis. Geographically, Tabuk Governorate is located along the north-west coast of KSA, closing to the Jordanian-Saudi Arabia border and facing Egypt across the Red Sea. It has a land area of 146.072 km^2^, 900 meters above sea level, and an Urban Area population of 687,000. Due to the ongoing Red Sea Project in this region, Tabuk has recently received a specific attention as one of the most working areas in KSA in attracting numerous workers from different nations. Moreover, Tabuk ranks as one of the biggest agricultural regions in the Kingdom with abundance of stagnant water and groundwater, palm trees, and wild herbs, and its weather is characterized with mild climate in the summer and cold and rainy climate in the winters, which collectively make it a good environment for the breeding of leishmaniasis´s insect (sandflies)-vectors.

**Study participants, data sources/measurement:** in this retrospective study, we used the surveillance database for the reported CL cases at the Vector-borne Diseases Control Unit, Local Health Directorate, Tabuk province, KSA. Data related to nationality, gender, age, and year-by-year and month-by-month distribution of the reported CL patients during the period from January 2006 to December 2021 were collected and analyzed. To meet the study objectives and the case definition, the included CL patients are defined as a person who was in Tabuk region during the said period and his/her CL was diagnosed by the standardized laboratory leishmaniasis-diagnosis as described by The WHO [22,23], and based on direct microscopic detection of leishmanial parasite (leishmanial amastigotes stages) in the Giemsa stained smears from patients´ skin scraping and biopsy specimens. In addition, the included CL patients had no repeated CL cases during the survey period.

**Statistical analysis:** data analysis were done using SPSS Statistics software package version 20.0 (SPSS Inc. Chicago, Illinois, USA). The Chi-square (χ^2^) test and Student “t” test or Mann-Whitney test were used for the categorical data and continuous variables as appropriate. A P-value of <0.05 was considered statistically significant.

*Funding sources:* this research did not receive any specific grant from funding agencies in the public, commercial, or not-for-profit sectors.

## Results

A total of 1575 CL patients, who were resident in the Tabuk region, KSA, during the period of 2006 to 2021, and were diagnosed and registered as CL patients, were analyzed here. The included CL patients had no repeated CL cases in their registers during the said period. Notably, as showed in Table 1, there was a continuous annual (year-by-year) record of these CL patients and ranged from 42 to 165 patient/year; reflecting the endemicity of the disease in the Tabuk region. Moreover, in terms of the recorded numbers, there was no significant variation by the nationality of these CL patients, but they showed significant distribution variations related to their gender, age group, and the annual and month-by-month record. As shown in Table 1, the registered 1575 CL patients included 836 (53.1%) and 739 (46.9%) Saudis and non-Saudi expatriates, respectively, with a total ratio around 1.1: 1.0. Of note, in years 2006, 2007, 2008, 2009, 2010, and 2011, the absolute number of these CL cases was higher in Saudis than in non-Saudis with a ratio around 1.3: 1.0, 1.4: 1.0, 1.6: 1.0, 1.2: 1.0, 1.1: 1.0, and 1.9: 1.0, respectively. However, by year 2017 to year 2021 the number of these CL cases was reversed between these Saudis and non-Saudis with a total ratio ranged from 0.9: 1.0 to 0.4: 1.0 (Table 1).

**Table 1:** number and distribution of the reported patients with cutaneous leishmaniasis (CL) in the Tabuk region, KSA, during the period from 2006 to 2021

Year	No. of cases	Nationality			Gender			Age group (years)				
		Saudis	Non-Saudi	Ratio	Male	Female	Ratio	<5	5≤10	10≤15	15≤45	≥45
2006	149*	83^&^	66	1.3:1.0	115^#^	34	3.3:1.0	16	24	21	73^†^	15
2007	165*	97^&^	68	1.4:1.0	124^#^	41	3.0:1.0	16	22	29	86^†^	12
2008	90	56^&^	34	1.6:1.0	66^#^	24	2.7:1.0	5	0	22	53^†^	10
2009	106	58^&^	48	1.2:1.0	93^#^	13	7.2:1.0	8	10	13	64^†^	11
2010	159*	83^&^	76	1.1:1.0	135^#^	24	5.6:1.0	13	15	14	95^†^	22
2011	125*	83^&^	42	1.9:1.0	91^#^	34	2.6:1.0	19	16	14	68^†^	8
2012	97	42	55^&^	0.7:1.0	88^#^	9	9.7:1.0	0	2	15	70^†^	10
2013	69	34	35	1.0:1.0	60^#^	9	6.6:1.0	4	15	10	40^†^	0
2014	68	35	33	1.1:1.0	63^#^	5	12.6:1.0	1	4	6	50^†^	7
2015	108	73^&^	35	2.0:1.0	80^#^	28	2.8:1.0	7	9	27	59^†^	6
2016	95	47	48	1.0:1.0	88^#^	7	12.5:1.0	5	1	11	67^†^	11
2017	97	46	51^&^	0.9:1.0	81^#^	16	5.1:1.0	2	6	8	73^†^	8
2018	90	41	49^&^	0.8:1.0	86^#^	4	21.5:1.0	3	2	10	69^†^	6
2019	65	28	37^&^	0.7:1.0	58^#^	7	8.3:1.0	0	0	2	56^†^	7
2020	42	16	26^&^	0.6:1.0	35^#^	7	5.0:1.0	2	0	0	37^†^	3
2021	50	14	36^&^	0.4:1.0	47^#^	3	15.6:1.0	2	0	0	42^†^	6
Total	1575	836	739	1.1:1.0	1310^#^	265	4.9:1.0^#^	103	126	202	1002^†^	142

* P <0.05 vs other years sets; ^&^P <0.05 vs the another nationality; ^#^P <0.05 vs females; ^†^P <0.05 vs other age group sets.

**Figure 1:**
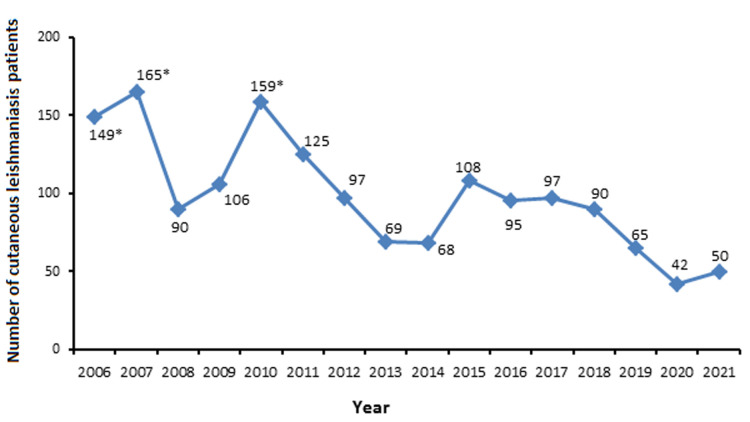
year-by-year distribution pattern of the reported patients with cutaneous leishmaniasis (CL) in the Tabuk region, KSA, during the period of 2006 to 2021

**Figure 2:**
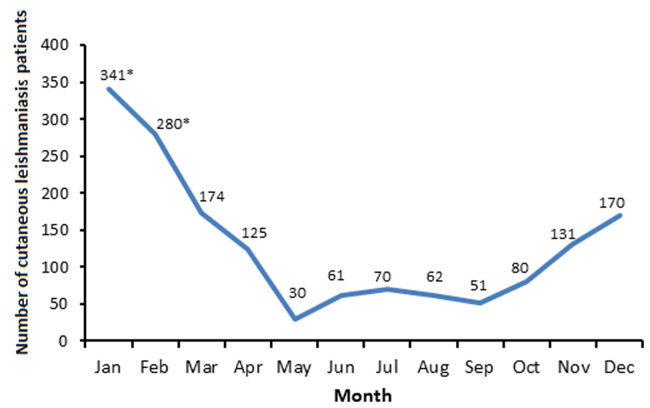
month-by-month distribution pattern of the reported patients with cutaneous leishmaniasis (CL) in the Tabuk region, KSA, during the period of 2006 to 2021

As represented in Table 1, the gender distribution of the reported 1575 CL patients showed that 1310 (83.17%) out of them were males while 265 (16.83%) were females. Moreover, the year-by-year recorded number of these CL patients was always significantly higher in males than in females, and the total male to female ratio was reached to 4.9: 1.0 (p <0.5) (Table 1). The age group distribution of these CL patients showed that their majority were in age group of 15-45 years (p <0.5), and the lowest number (103/1575; 6.5%) was in age group of <5 years Table 1). Moreover, year-by-year record number of these CL patients was always significantly (p <0.5) higher in those with age group of 15-45 years (Table 1). As demonstrated in Table 1 and Figure 1, though there was a continuous annual record of these CL patients that reflected the endemicity of the disease in the Tabuk region, the year-by-year recorded number of these CL patients was to reach its peaks levels in 2006, 2007, 2010, and 2011 (149, 165, 159, and 125 CL patient, respectively), and the lowest number (42 CL patient) was reported in 2020 (Table 1, Figure 1). Furthermore, month-by-month distribution variation was also observed here in the annual record of these CL patients, whereby their highest numbers were reported in the January and February compared to the rest of the year months (Figure 2).

## Discussion

Cutaneous leishmaniasis (CL); the most common clinical form of leishmanial diseases worldwide [7,24,25], is lately classified by the WHO as one of the most neglected tropical diseases for which sustainable epidemiological investigations and monitoring disease trends is a priority [16,17]. The present study was therefore designed to reveal the epidemiological trend of CL in the Tabuk province, KSA, over the period of 2006-2021. A total of 1575 Saudi and Non-Saudi patients of both sexes and different ages, who had CL and were resident in the Tabuk region, were detected and registered during this period. In addition, there was a continuous annual and month-by-month record of these CL patients; reflecting the disease endemicity in this region.

The burden of leishmaniasis, particularly its CL form, continues to be one of the major health problems in many regions of KSA, and the endemicity of the disease was reported in the Eastern, Riyadh, Al-Hassa, Aseer, Al-Qaseem, Ha'il, and Jazan regions of the country [19-21]. In this circumstance, Tabuk is one of the biggest agricultural regions in KSA. Rapid urbanization, climate changes, and invasion of disease reservoirs by the humans movements, particularly the movements of foreign workers from leishmaniasis endemic areas in the neighboring countries, might robustly link to the transmission, spatiotemporal distribution, and persistence of leishmaniasis in this region [19,22,26,27]. Tabuk region has abundance of stagnant water, groundwater, irrigation schemes, palm trees, and wild herb, that collectively prone it an enriching environment for the breeding and survival of leishmaniasis´s insect-vectors [22,26,27]. Furthermore, the Syrian war and its related significant displacement of refugees into several neighboring countries might also lead to increased frightening numbers of CL cases across these neighboring countries, including KSA [5,17].

The present study focused on whether there were distribution variables among the reported CL patients related to their nationality, age, gender, and the annual and month-by-month records. The total results showed no significant difference related to the patients' nationality. On the other hand, gender-and-age-dependent variations were significantly observed here whereby the majority of the reported CL patients were in those with age group of 15-45 years, the disease affected males more than females. Most importantly, a continuous annual and month-by-month record of these CL patients; as an evidence of CL endemicity in this region, as well as the annual and month-by-month record variations, were also detected. In agreement with the present findings, various earlier studies have been conducted in the different regions of KSA and reported that the CL disease affected males more than females, and the younger aged and adult individuals more than the other group of ages, but there was no significant variation in terms of number of CL cases between the Saudis and non-Saudi expatriates [27,28]. The observed gender-based distribution among the reported CL patients could be attributed to the fact that the males are more exposed to the parasite insect vectors as a result of their occupational activities [29], and the women in Saudi Arabia often have limited time facing to these vectors as they're often indoors staying for long time, and they cover almost their whole body when outdoor [19]. Moreover, the majority of the reported CL patients were in the 15 years and above age group, and the lowest number was in age group of <5 years. These findings are in constancy with those reported worldwide [30,31]. However, CL tends to more affect younger age group has also been reported in some leishmaniasis endemic areas [32-34]. Furthermore, the annual and month-by-month variations of the CL incidence rates were also reported; and such seasonal emergence, declining, and re-emergence trends of the disease might be related to several factors such as variabilities in breeding activity and survivability of sandflies insect vectors [26-28], changes in the public health approaches [35], and changes in the practices, behaviors and perceptions related to CL [36].

**Study strengths and limitations:** the present survey included a number of important variables such as the annual and month-by-month distribution of the reported CL patients and variations in terms of their number and their residency, gender, and specific age group. Nevertheless, it would have been preferable if additional factors were available in the records of these CL patients and are included here; such as variables related to their general clinical data, occupational hazardous, anatomical distribution and morphological typing (popular, nodular, or ulcerative) of the detected CL, molecular (PCR) typing of the underlying causative *Leishmani* species, and type and outcome of the applied treatment.

## Conclusion

Data of the present study were drawn from the registers of the Saudi and non-Saudi patients with CL; who were resident (2006-2021) in the Tabuk region, KSA, and revealed the endemicity of this protozoan parasitic disease in this region. Further screening implements and large-scale monitoring studies are warranted to assess the entire epidemiological features of CL disease, and to improve the public health measures to thorough control its endemicity in KSA.

### What is known about this topic

- *There are scarce evidence in the literature related to the epidemiological trends of cutaneous leishmaniasis (CL) in the Tabuk region, Saudi Arabia;*
- *The leishmaniasis´s control program in KSA remains challenging*.

### What this study adds

- *The present findings provide an insight into the endemicity of CL in the Tabuk region of KSA;*
- *The present findings raise the essentiality for improving the controlling polices against Leishmania infections and their related diseases in KSA*.
